# Creating building-level, three-dimensional digital models of historic urban neighborhoods from Sanborn Fire Insurance maps using machine learning

**DOI:** 10.1371/journal.pone.0286340

**Published:** 2023-06-28

**Authors:** Yue Lin, Jialin Li, Adam Porr, Gerika Logan, Ningchuan Xiao, Harvey J. Miller

**Affiliations:** 1 Department of Geography, The Ohio State University, Columbus, Ohio, United States of America; 2 Center for Urban and Regional Analysis, The Ohio State University, Columbus, Ohio, United States of America; 3 Epsilon, Chicago, Illinois, United States of America; 4 Mid-Ohio Regional Planning Commission, Columbus, Ohio, United States of America; Chulalongkorn University, THAILAND

## Abstract

Sanborn Fire Insurance maps contain a wealth of building-level information about U.S. cities dating back to the late 19th century. They are a valuable resource for studying changes in urban environments, such as the legacy of urban highway construction and urban renewal in the 20th century. However, it is a challenge to automatically extract the building-level information effectively and efficiently from Sanborn maps because of the large number of map entities and the lack of appropriate computational methods to detect these entities. This paper contributes to a scalable workflow that utilizes machine learning to identify building footprints and associated properties on Sanborn maps. This information can be effectively applied to create 3D visualization of historic urban neighborhoods and inform urban changes. We demonstrate our methods using Sanborn maps for two neighborhoods in Columbus, Ohio, USA that were bisected by highway construction in the 1960s. Quantitative and visual analysis of the results suggest high accuracy of the extracted building-level information, with an F-1 score of 0.9 for building footprints and construction materials, and over 0.7 for building utilizations and numbers of stories. We also illustrate how to visualize pre-highway neighborhoods.

## 1 Introduction

Cities in the United States have undergone dramatic changes in the 20th century. The development of the streetcar and the personal automobile profoundly altered the millennia-old urban development patterns that were constrained by walking as the primary mode of transport. In the mid to late 20th century, construction of urban highways combined with Federal support for mortgage lending favoring new construction helped to encourage widespread suburbanization, partly through the Federal-Aid Highway Act (1956), continuing the drop in population densities in central cities [1]. Much of this suburbanization was selective, favoring White people over Black and Brown people due to exclusionary zoning, racist deed covenants and other blatantly discriminatory practices in the suburbs. Those remaining in central cities suffered from neighborhood disinvestment due to redlining practices that restricted mortgage lending to communities of color [2]. Adding to this stress and disinvestment was the aforementioned urban highway construction that damaged, disconnected and in some cases completely destroyed vulnerable neighborhoods, and urban renewal projects that replaced historic structures with modernist buildings, parking lots, and housing projects [3, 4].

The negative legacies of 20th century urban development practices persist to this day: many urban neighborhoods with low social and health outcomes are the same neighborhoods that were redlined and suffered from highway construction [5, 6]. The altered built environments also exacerbate urban heat island effects due to the overabundance of concrete and asphalt [7]. Recognition of these persistent harms has generated interest in reconstructing built environments that were damaged and destroyed in the late 20th century; this can support research into environmental and social history, facilitate outreach and engagement, and guide policy and design prescriptions, such as the removal of urban highways and the reshaping of the urban fabric [8].

In recent years, geo-humanities have emerged as a research field where geographic science and the humanities converge, in which researchers are increasingly interested in methods that can be used to enhance the ways we research, disseminate, and interpret the history of urban environments [9]. Historical maps are a valuable resource for geo-humanities research because they often contain retrospective geographic information that can be difficult to find elsewhere [10–15]. Among the many historical maps, Sanborn Fire Insurance maps provide highly detailed historic building-level urban information in over 12,000 American cities and towns dating back to the 19th century [16, 17] (see a more detailed discussion in the Background section). These maps are now available in digital format through various online archives [18], such as the digital collection curated by the Library of Congress (https://www.loc.gov/collections/sanborn-maps/about-this-collection/), providing researchers with a valuable resource to study the evolution of urban landscapes over time.

Extracting information from the Sanborn maps is challenging because the information contained in these maps (e.g., building footprints) is not designed to be machine readable and is difficult to manage in a structured database format. Sanborn maps were lithographically printed and hand-colored with waxed paper stencils [19]. A conventional method for extracting geographic information from Sanborn maps is to manually georeference and label map entities using geographic information systems (GIS) software such as ArcGIS Pro and QGIS [20–22]. This manual method is limited because each map contains a large amount of information to be processed. Over the past decade, the rapid advancement of machine learning techniques has facilitated the development of automated and semi-automated workflows for extracting geographic information from historical maps [10, 23–25] (see a more detailed discussion in the Background section). Methods have been developed to efficiently detect textual labels textual labels [26–28], land use [29, 30], building footprints [31, 32], road networks [33–36], and landmarks [37, 38]. Existing methods, however, are mostly focused on maps other than Sanborn maps, such as the topographic maps from the United States Geological Survey (https://www.usgs.gov/programs/national-geospatial-program/historical-topographic-maps-preserving-past), which contain different types of geographic information and use different symbol and color systems than the Sanborn maps. These methods may not be directly applicable to Sanborn maps. Although there is literature that uses machine learning to extract information from Sanborn maps [37], these methods are limited to specific types of buildings, such as manufactured gas production and storage sites, and are difficult to generalize to other information on Sanborn maps (e.g., building footprints of dwellings and stores). It is still difficult to create efficient workflows for extracting building-level information from Sanborn maps.

In this paper, we address the limitations of existing studies by presenting a scalable workflow for extracting geographic information from Sanborn maps. We focus specifically on building footprints and associated properties (construction materials, utilizations, and numbers of stories). Buildings are the base level fabric of a city, and 3D historic building data can support measurement, analysis, and understanding of how neighborhood environmental, social, and health conditions, as well as lifestyles, have changed over time in cities. This can also help with the development of high-fidelity 3D visualizations and virtual reality experiences of historic neighborhoods, supporting education, outreach, and engagement on urban history, including the shocks and disruptions of 20th century urban policy, development, and infrastructure projects. The ability to generate these data at scale for U.S. cities can support research and outreach at scale of an entire city, over time, and comparisons across cities at the national scale.

The Background section provides a comprehensive overview of the Sanborn maps and how machine learning can be utilized to analyze historical maps. The Methods section details the proposed workflow. The Application section examine the effectiveness of the workflow by applying it to reconstruct the historic neighborhoods of Hanford Village and Driving Park in Columbus, Ohio, USA that suffered from urban renewal and highway construction in the 20th century. The Discussion and Conclusions section provides a discussion of its limitations and potential future research.

## 2 Background

Historical maps, typically available through online map archives and libraries today, provide valuable insights into the past. In the United States, early historical maps from the colonial and early national periods depict the first explorations and settlements, the boundaries of colonial territories, and the wars and military activities that led to independence [39, 40]. These maps are often limited in geographic knowledge and accuracy, reflecting the lack of information about the land and resources available. From the mid-19th century onwards, professionally surveyed historical maps such as topographical, railroad, canal, city plans, and insurance maps emerged, which illustrate the westward expansion of the United States and the development of its transportation and housing infrastructure [17, 41]. Unlike the earlier maps, these professionally surveyed maps provide detailed and reliable geographic information and are valuable for comprehending the changing social and environmental landscapes of the United States over time.

### 2.1 Sanborn maps

**Fig 1:**
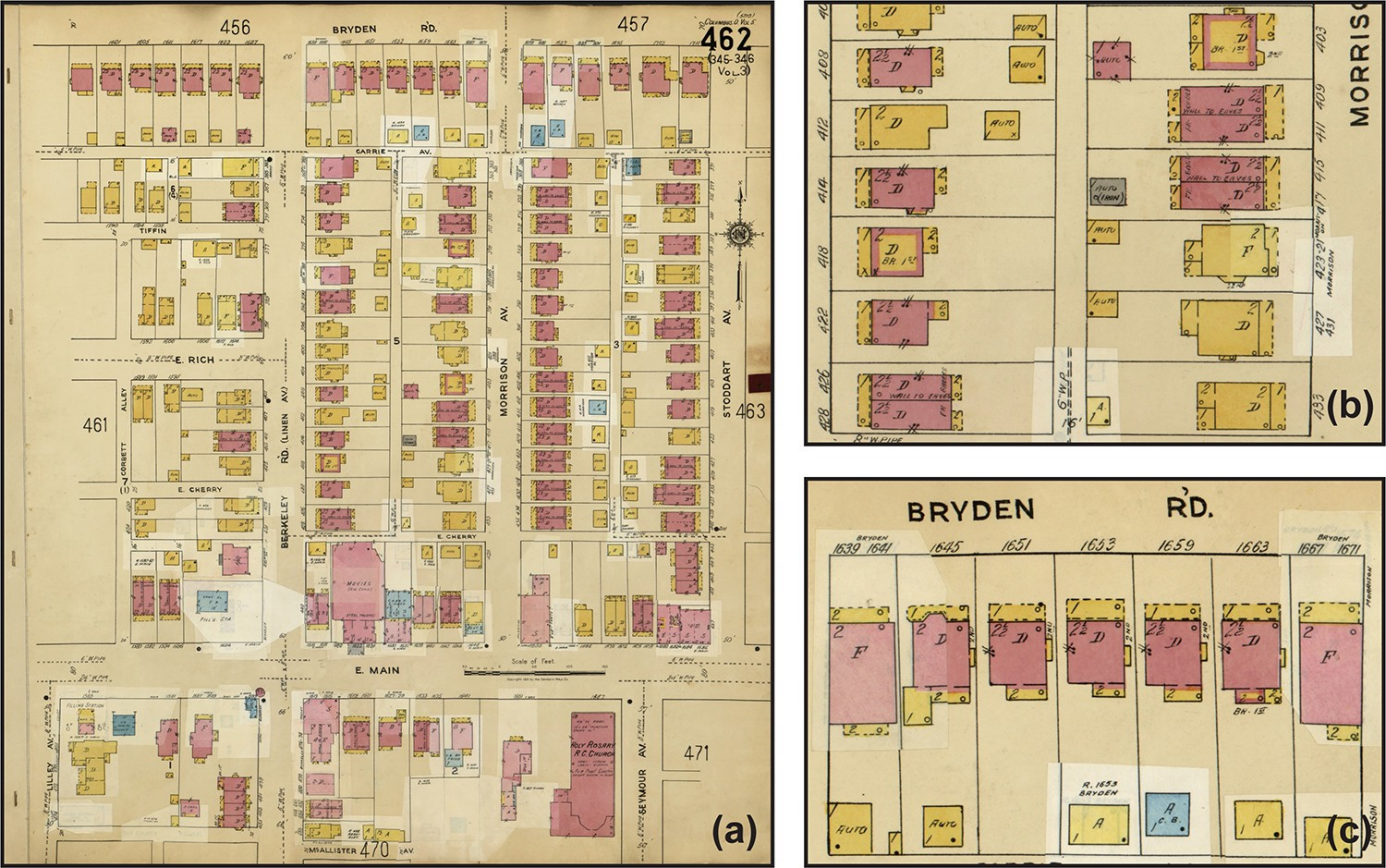
A 1961 Sanborn map for Hanford Village in Columbus, Ohio. (a) is the full Sanborn map sheet. Inset maps (b and c) show the details of the building-level information. Polygons shaded in yellow, pink, blue, and gray are the footprints of various buildings where the colors represent the materials used in building constructions. The abbreviated labels “D”, “F”, and “Auto” in each polygon represent dwelling, flat, and automobile, respectively. The numerals “1”, “2”, and “2 ½” in each building represent the number of stories.

Among the many historical maps, Sanborn maps are an excellent source of highly detailed historic building-level urban information [16, 17]. Originally created in the late 19th and early 20th centuries to evaluate fire insurance liability, Sanborn maps have been continuously produced through today and covered more than 12,000 American cities and towns. Atlas pages contain information such as street names, parcel boundaries, block numbers, building footprints, as well as the construction materials, utilization, and number of stories of each building (Fig 1). Digital scans of Sanborn maps are now widely available in a variety of online archives [18], including the Library of Congress’s digital collection, which enables the analysis of Sanborn maps on a large scale.

Sanborn maps are considered valuable historical documents in the humanities and social sciences that have been extensively used by historians, architects, and urban planners to study the development of cities and towns over time. For example, Sanborn maps are of considerable value for tracking changes in urban land use, providing insights into the impact of industrialization and urbanization on the natural environment [12, 42–44]. Sanborn maps are also an important tool for investigating the development and evolution of urban morphology [45–49]. By analyzing the changes in key physical elements that shape the urban environment, such as streets, public spaces, and buildings, Sanborn maps can provide insights into the shifting patterns of urban development, demolition, and redevelopment experienced by many U.S. cities. Sanborn maps are also useful in evaluating the impact of various factors such as natural disasters [50], depopulation [51], urban renewal [52], and railroad abandonment [53] on urban morphology and the physical, social, and economic dimensions of urban change across different historical periods. Due to the level of effort required to extract features and attributes manually from the maps, such analyses are often limited to small geographic study areas and limited sets of attributes. Automated feature extraction may someday allow for inexpensive bulk processing of entire collections of Sanborn maps, resulting in a database that covers communities throughout North America and spanning many decades. Such an expansive collection of building data would allow for detailed spatiotemporal comparison of indicators related to the built environment such as footprint area, floor-area ratio, setback distances, prevalence of architectural styles, mixing of use classes, and conversion of uses from one class to another. These indicators could be integrated with other indicators derived from large-scale land use [54], transportation [55], and economic [56] data, for example, to better understand the complex relationships between the built environment and other factors.

Creating immersive and interactive 3D digital models using information from Sanborn maps can be highly beneficial in the applications of these maps within the humanities and social sciences. These models offer realistic 3D representations of past urban environments that can be compared across different time periods, and even contrasted with present-day urban landscapes, which can provide scholars and planners with an intuitive and comprehensive understanding of what has been lost or gained during urban development and its lasting implications [52, 57]. In addition, these 3D models enable 3D analytics of past built environments, such as visibility analysis of historic buildings [58], which reveals how the evolution of cities and towns affects quality of life and uncover important patterns and relationships that may not be readily apparent in non-spatial data or 2D maps.

### 2.2 Analyzing historical maps using machine learning

Over the past decade, advancements in machine learning techniques have brought significant improvements to various computer vision applications, including the analysis of historical maps. These techniques have greatly facilitated the automated processing and understanding of historical maps on a large scale, resulting in three primary developments in historical map analysis. First, machine learning algorithms such as support vector machine (SVMs), multilayer perceptrons (MLPs), and convolutional neural networks (CNNs) have been developed to classify historical maps based on their features, including scale, style, projection, and content, which enables building map databases with rich metadata that can be used for research and analysis [23, 25]. Second, methods such as generative adversarial networks (GANs) have been used to transfer the style of one map to another, allowing for the creation of new maps that combine the content of historical maps with modern design elements [59]. Third, optical character recognition (OCR), a commonly used method for detecting textual labels on historical maps [26–28], and machine learning techniques such as CNNs have been applied to detect and extract information from historical maps, including specific features such as textual labels [26–28], land use [29, 30], building footprints [31, 32], road networks [33–36], and landmarks [37, 38]. This information can be used for various purposes, including urban planning, cultural heritage preservation, and historical research.

The progress in machine learning techniques for historical map analysis holds great potential to efficiently extract geographic information such as building footprints and associated properties from Sanborn maps to create realistic 3D digital models of historic urban environments. However, it is crucial to recognize that these techniques cannot be directly applied to Sanborn maps as they are designed for other types of historical maps that have vastly different map elements and representations. For example, the current methods for building footprint detection are developed mainly for topographical maps [31, 32], where buildings are represented as small rectangles with solid black color, unlike in Sanborn maps, where buildings are depicted as polygons with varying shapes and colors. Applying these methods directly to Sanborn maps is therefore not feasible. Regarding identifying building properties on Sanborn maps, OCR [26–28] may be employed to extract building properties such as utilization and the number of stories that are labeled using letters, words, and numerals. However, since the labels on Sanborn maps are in handwriting styles, existing OCR techniques, which are typically designed to detect printed text, may result in low accuracy in textual detection for Sanborn maps [60]. In addition, most CNN-based models for text detection are trained on railroad [61] or topographical maps [62] instead of labeled textual data from Sanborn maps, and thus applying existing CNN-based models to detect building properties on Sanborn maps may also be ineffective. Therefore, it is necessary to develop new models or train existing ones specifically for Sanborn maps to leverage the progress in machine learning for extracting information from Sanborn maps effectively.

## 3 Methods

**Fig 2:**
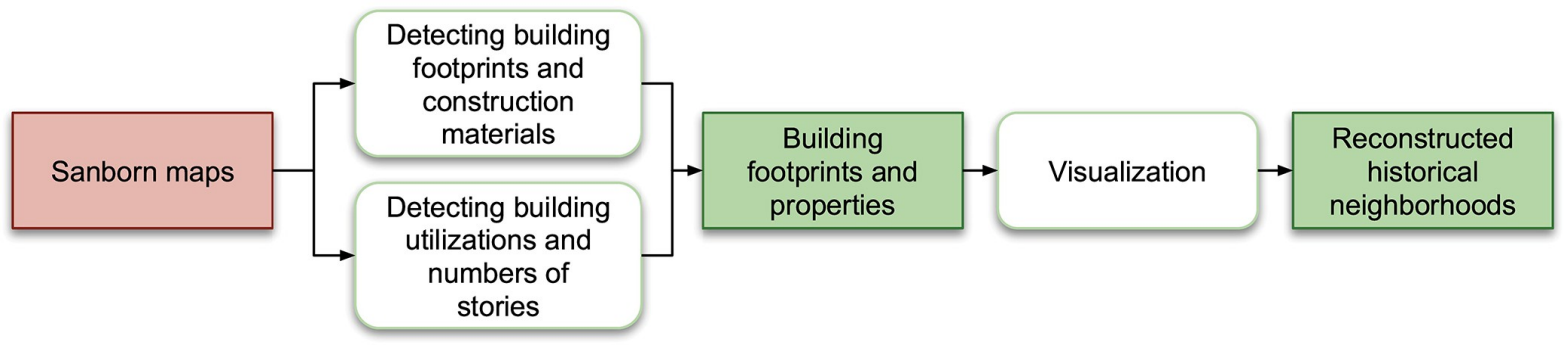
A flowchart of the proposed workflow. An orange-pink rectangle indicates input data, an open rounded rectangle indicates an operation, and a green rectangle represents intermediate or final output.

Fig 2 illustrates the workflow we develop to extract building footprints and associated properties. The workflow begins with Sanborn maps, upon which we develop machine learning techniques to detect the building footprints and associated properties. This information can then be utilized to generate 3D digital models, enabling us to visualize the historic neighborhoods with great detail and accuracy.

### 3.1 Detecting building footprints and construction materials

Sanborn maps contain building footprints as polygons of various shapes. The building footprint fill color indicates the materials used in their construction, and these colors distinguish them from the beige background and other symbols and text that are printed in black (Fig 1). To detect building footprints and construction materials on Sanborn maps, we develop a workflow that includes two steps: pixel-based classification and post-processing. Pixel-based classification involves training and evaluating a machine learning model to classify each pixel as belonging to a non-building (e.g., background or text) or a building made of one of the five construction materials (Table 1). Postprocessing converts groupings of pixels into building objects and refined building outlines. Our approach is well-suited for detecting building footprints and construction materials on Sanborn maps compared to completely object-based detection approaches such as the Mask R-CNN [63], as it is relatively straightforward to implement and can produce effective results. Specifically, pixel-based classification is relatively straightforward and does not require building complex deep neural network models. In addition, it typically only requires labeling a limited number of regions of interest in the image, rather than a large number of building footprints for training and testing. Our approach also benefits from the distinct color patterns used for buildings on Sanborn maps, which are clearly distinguishable from the background and non-building features.

**Table 1:** Building construction materials and the colors that distinguish them on Sanborn maps.

Class	Description	Color
**1**	Brick or tile building	Reddish/pink
**2**	Frame or wood building	Yellow
**3**	Fire resistive building	Olive green
**4**	Adobe, metal, or iron building	Gray
**5**	Concrete or cinder block building	Blue
**6**	Non-building: background	Beige
**7**	Non-building: other symbols or text	Black

#### 3.1.1 Pixel-based classification

Data prepared using pixels from Sanborn maps are needed to train and evaluate the model for pixel-based classification. These pixels should represent both non-buildings and buildings made of various materials on Sanborn maps. For each pixel, we obtain its RGB (red, green, and blue) values and manually categorize it into one of the seven classes in Table 1. This process is known as labeling. We then divide the labeled pixels into two data sets, *P*1 and *P*2, where *P*1 is used for model training and *P*2 for model evaluation.

We use a SVM [64] for pixel-based classification. SVMs are a class of supervised machine learning models that have been used in a variety of classification tasks such as sentiment analysis [65] and spam detection [66]. It has also been widely applied in remote sensing [67, 68] and image analysis applications [69] due to its effectiveness in handling high-dimensional and non-linear data, and their ability to provide accurate results with relatively simple implementation. The objective of a SVM is to find an optimal hyperplane that best separates different classes in a feature space with *n* dimensions that correspond to *n* explanatory variables. An optimal hyperplane has the largest distance to the closest data point of any class in the feature space. This process is known as training. In this study, we use some of the labeled pixels to train a SVM to find an optimal hyperplane that distinguishes between different classes of pixels in a feature space with three dimensions of RGB (red, green, and blue).

We evaluate the accuracy of the trained model using three metrics: precision, recall, and F-score. We refer to this as pixel-level accuracy evaluation because the purpose is to examine the model accuracy in classifying each pixel. To calculate these accuracy metrics, we define a 7×7 matrix **M** = {*m*_ij_}, where each element *m*_ij_ denotes the number of pixels in class *i* that are categorized as class *j* by the model. We have assigned classes 1 through 5 to buildings, therefore precision is the proportion of correctly classified building pixels in class *i* (1 ≤ *i* ≤ 5) among all pixels assigned to class *i* by the model:

$$\mathrm{Precision}=\frac{m_{ii}}{\sum_{j}m_{ji}}$$ (1)

Recall is the proportion of correctly classified building pixels in class *i* (1 ≤ *i* ≤ 5) among all pixels manually labeled in class *i*:

$$\mathrm{Recall}=\frac{m_{ii}}{\sum_{j}m_{ij}}$$ (2)

F-score is the harmonic mean of precision and recall:

$$F‐\mathrm{score}=2\frac{\mathrm{Precision}\times \mathrm{Recall}}{\mathrm{Precision}+\mathrm{Recall}}$$ (3)

**Fig 3:**
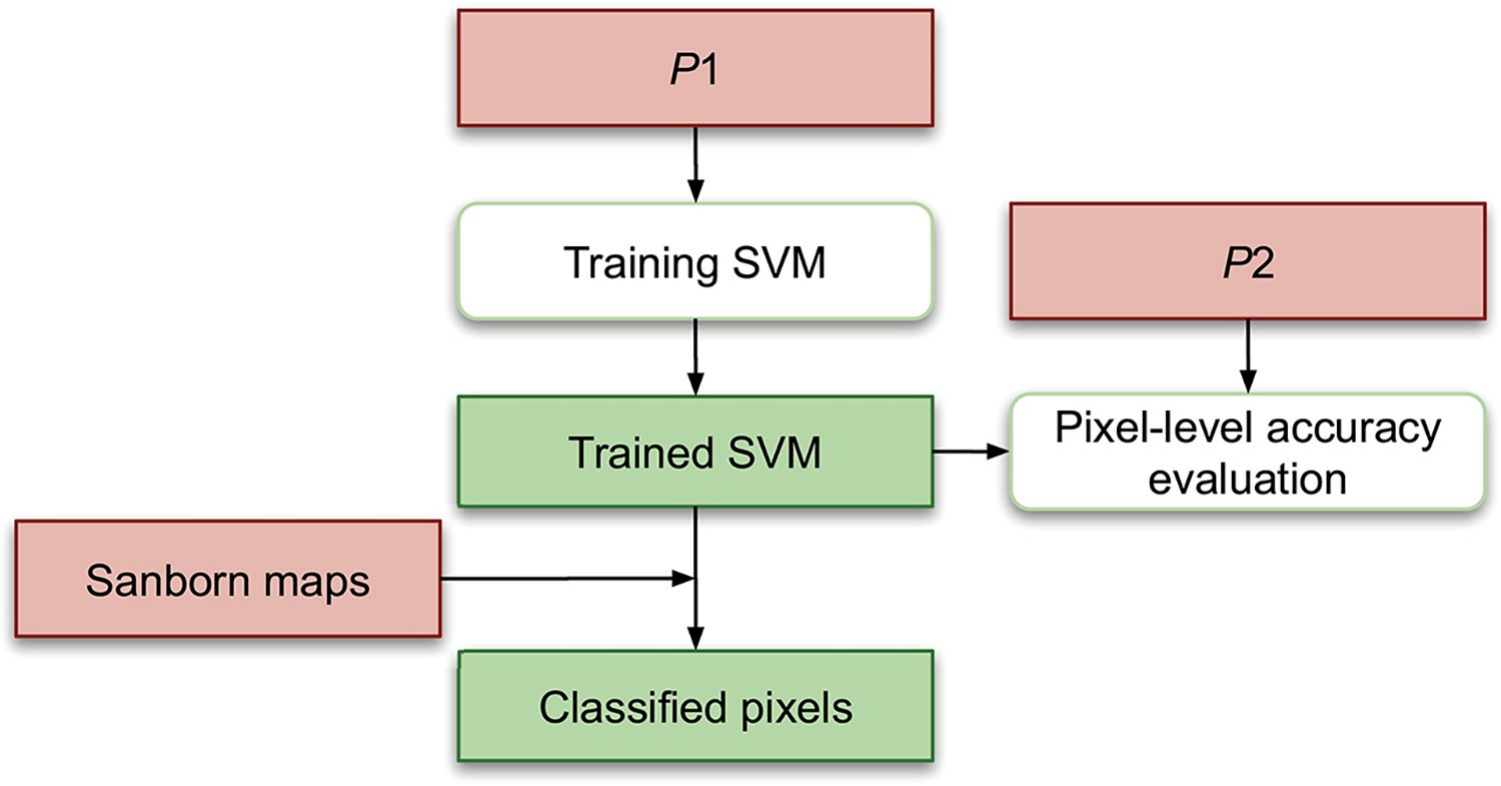
Pixel-based classification. An orange-pink rectangle indicates input data, an open rounded rectangle indicates an operation, and a green rectangle represents intermediate or final output.

Fig 3 presents the training and evaluation of a SVM for pixel-based classification. We use data set *P*1 to train the SVM, and evaluate the trained model on data set *P*2 using precision, recall, and F-score. After training and evaluating the SVM, we input the Sanborn maps into the trained model to classify each pixel on the maps.

#### 3.1.2 Postprocessing

We post-process the classified pixels on Sanborn maps in four steps to produce vector GIS buildings objects with refined boundaries that are suitable for reconstructing 3D building objects. First, morphological transformations [70] remove noise and close small holes inside buildings based on the operations of dilation and erosion. Dilation adds pixels to region boundaries, while erosion removes pixels from the boundaries. Erosion followed by dilation is *opening*: this eliminates thin protrusions, removing noise. Dilation followed by erosion is *closing*: this fills small holes and gaps in buildings. The second step is vectorization, which converts building pixels to objects by generating polygons that encompass connected pixels classified as belonging to buildings with the same materials. The third step is simplification [71], which simplifies building objects by removing small details and corridors. Finally, we perform regularization [72] to ensure that building objects are comprised of right angles or diagonals, or are circular in shape.

We evaluate the accuracy of the detected building objects using a data set called *O*1. We generate this data set by manually dividing each Sanborn map into multiple non-overlapping tiles and selecting a subset of these tiles at random. We manually identify building footprints from the selected map tiles, and each building has a manual label that indicates the material used in its construction. Using tiles is necessary as labeling all buildings on every map sheet would be time-consuming. Although one might suggest evaluating using buildings on only one map sheet, it carries the risk of lacking diversity in the test data set as buildings from one sheet may not represent the entire study area. We describe how map tiles are created in the third section. To determine if a detection is correct, we first collect all buildings (both detected and manually labeled) in each map tile from *O*1. We compare the footprint of each detected building *F*_d_ to that of its nearest labeled building made of the same construction material, denoted as *F*_l_. We compute a metric called intersection over union (IoU) as the ratio of the intersection area of *F*_d_ and *F*_l_ to their union area:

$$\mathrm{IoU}=\frac{\mathrm{area}(F_{d}\cap F_{l})}{\mathrm{area}(F_{d}\cup F_{l})}$$ (4)

When the IoU value for a building in class *i* exceeds 0.5, more than half of the detected footprint overlaps with the footprint of a building in the same class that is considered as its ground truth [73]. In this case, it is reasonable to state that this building in class *i* is correctly detected.

We apply similar metrics—precision, recall, and F-score—as used for pixel-based accuracy evaluation to assess the accuracy of the detected building objects. Precision is the proportion of correctly detected building objects in class *i* (1 ≤ *i* ≤ 5) among all objects detected in class *i*. Recall is the proportion of correctly detected building objects in class *i* (1 ≤ *i* ≤ 5) among all objects labeled as class *i*. F-score is the harmonic mean of precision and recall.

**Fig 4:**
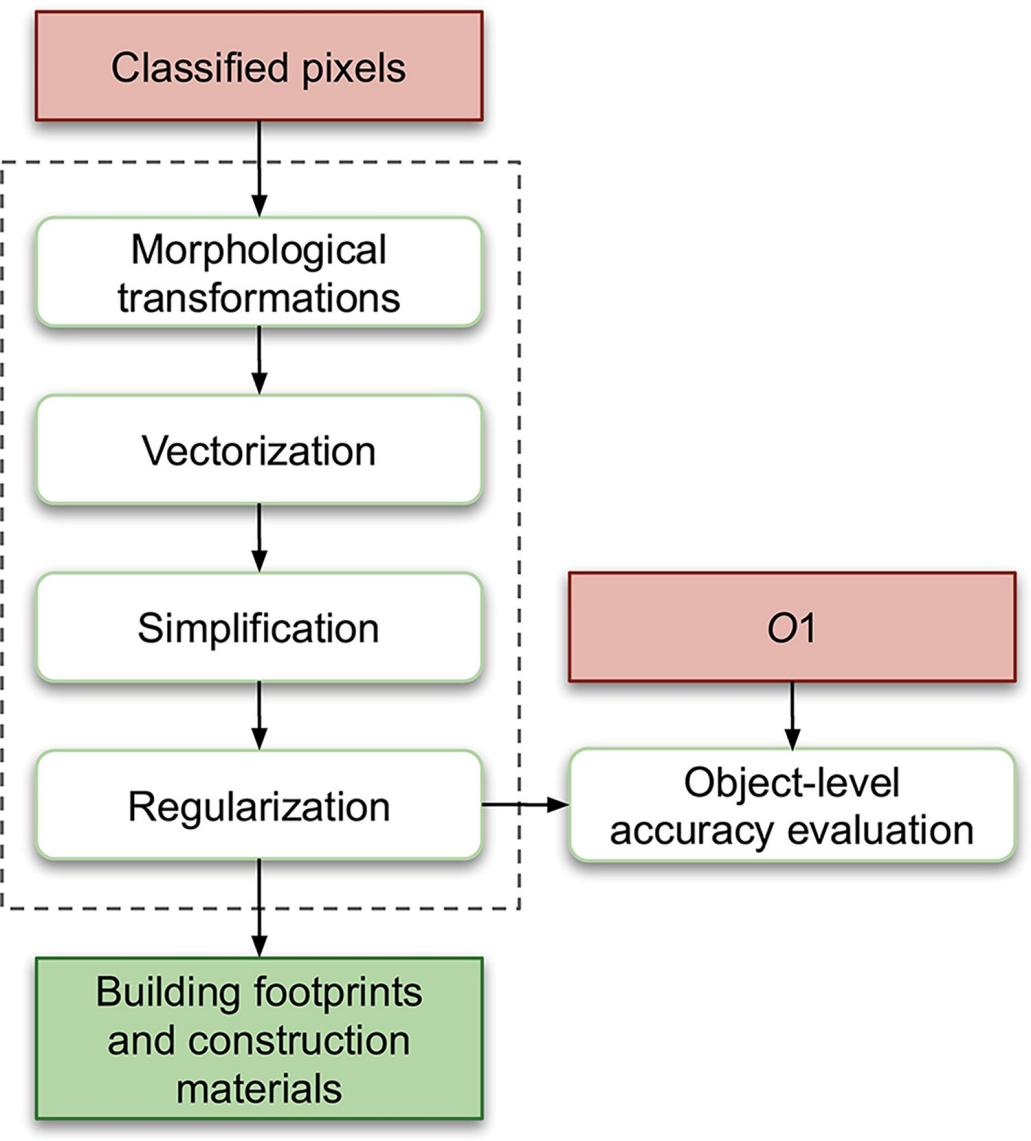
Postprocessing. An orange-pink rectangle indicates input data, an open rounded rectangle indicates an operation, and a green rectangle represents intermediate or final output.

Fig 4 illustrates the postprocessing process and its evaluation. We postprocess the classified pixels output from the Pixel-Based Classification section through four steps (morphological transformations, vectorization, simplification, and regularization) to obtain vector GIS building objects that indicate building footprints and construction materials. We evaluate the accuracy of these building objects on data set *O*1, and we refer to this evaluation as object-level accuracy evaluation.

### 3.2 Detecting building utilizations and numbers of stories

On the Sanborn maps, abbreviations within each building denote building properties such as utilizations and numbers of stories. The utilization of a building is abbreviated to a letter or word (examples shown in Table 2), and the number of stories is represented as a numeral (e.g., “1”, “1 ½”, or “2”). Fig 1 illustrates these abbreviations on Sanborn maps.

**Table 2:** Examples of letters and words as abbreviations of building utilizations.

Letter or word	Description
**A**	Automobile (or a garage)
**Auto**	Automobile (or a garage)
**D**	Dwelling
**F**	Flat (or an apartment)
**S**	Store
**PO**	Post office

A comprehensive glossary containing 302 abbreviated building utilizations is available at the California State University Northridge Map Library [74].

We prepare four data sets to train and evaluate two deep learning models for detecting building utilizations and numbers of stories, respectively. We use the first data set, denoted as *U*1, to train a machine learning model for building utilization detection. It contains a subset of non-overlapping map tiles from Sanborn maps that cover buildings of various utilizations, and we process these map tiles through manually identifying the bounding box of each abbreviated letter or word that denotes a building utilization. We evaluate the model trained on *U*1 using a second data set called *U*2, which contains a different subset of map tiles with manually labeled bounding boxes of abbreviated letters and words. We use the third data set, *S*1, to train a machine learning model for detecting numbers of stories, and this data set consists of map tiles covering various numerals representing different possible numbers of stories. We manually identify the bounding box of each numeral. The fourth data set, *S*2, evaluates the model trained on *S*1. This data set contains different map tiles from *S*1 with all numerals representing numbers of stories manually labeled.

**Fig 5:**
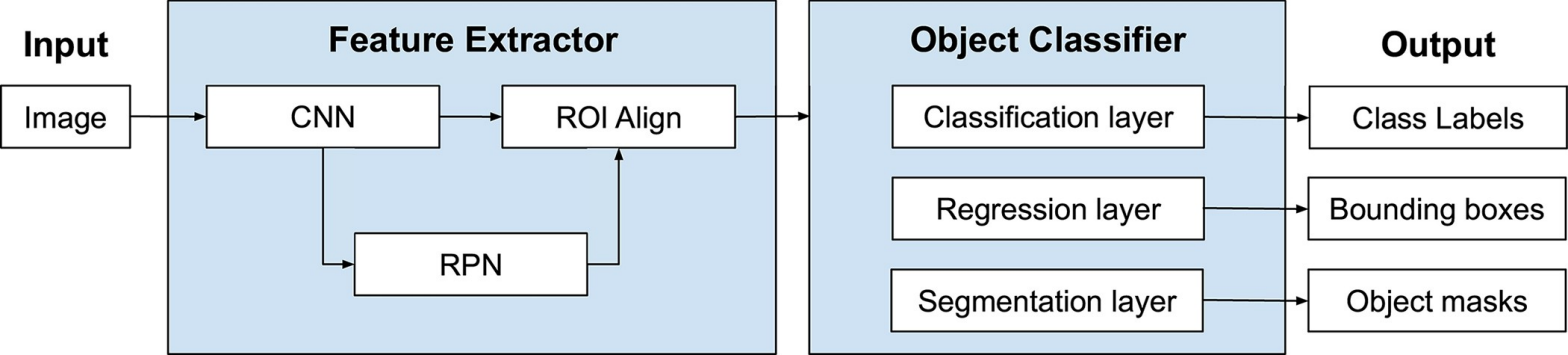
Network structure of Mask R-CNN.

We develop two machine learning models, denoted as *M*1 and *M*2, for detecting building utilizations and numbers of stories, respectively. Both *M*1 and *M*2 are based on the Mask R-CNN model [63], which is a region-based convolutional neural network (R-CNN) that is widely used for text recognition [75, 76]. The Mask R-CNN model comprises two stages, illustrated in Fig 5. In the first stage, a backbone CNN and a region proposal network (RPN) are used to predict regions of interest (ROIs) that may contain the target objects (in our case, abbreviations). These ROIs are processed through the ROI Align layer to make them the same size as input to the second stage. In the second stage, the model predicts the class labels, bounding boxes, and object masks for each predicted ROI from the first stage. A confidence score is associated with the class label to indicate the probability of correct classification.

We evaluate the accuracy of the two machine learning models built on Mask R-CNN using a metric called average precision (AP) [73]. The evaluation begins by listing all the detected objects on each map tile from the test data set (*U*2 or *S*2), where we sort the detected objects by their confidence scores in a descending order. We compare the bounding box of each detected object to that of its nearest manually labeled object, and calculate the intersection over union (IoU) as the ratio of their intersection area to their union area. An IoU value greater than 0.5 indicates that the detection is correct. For every position (or rank) of the sorted list of detected objects, we define precision as the proportion of correctly detected objects in class *i* among all objects detected in class *i* above the given rank. We define recall as the proportion of correctly detected objects in class *i* above the given rank among all objects labeled as class *i*. Let R = {*r*_1_, *r*_2_,…, *r*_N_} be a set of equally spaced recall levels with *r*_1_ = 0, *r*_N_ = 1, and *r*_k_ ≤ *r*_k+1_ (1 ≤ *k* < *N*), *p*_i_(*r*_k_) the precision at recall level *r*_k_ for class *i*, and *p*_i_,_max_(*r*_k_) = max{*p*_*i*_(*r*_j_), *j* ≥ *k*} the maximum precision at recall levels *r*_k_ for class *i* (i.e., the maximum precision at recall levels exceeding or equal to *r*_k_ for class *i*). The AP of class *i* is the average of maximum precisions at the *N* recall levels in *R*:

$${AP}_{i}=\frac{1}{N}\sum_{r\in R}p_{i,\max}(r_{k})$$ (5)

The value of AP_i_ ranges from 0 to 1, with 1 indicating that all objects in class *i* are correctly detected. In practice, the value of *N* is often set to 11 and thus *R* = {0, 0.1, …, 1} [73]. This setting is adopted in this paper.

**Fig 6:**
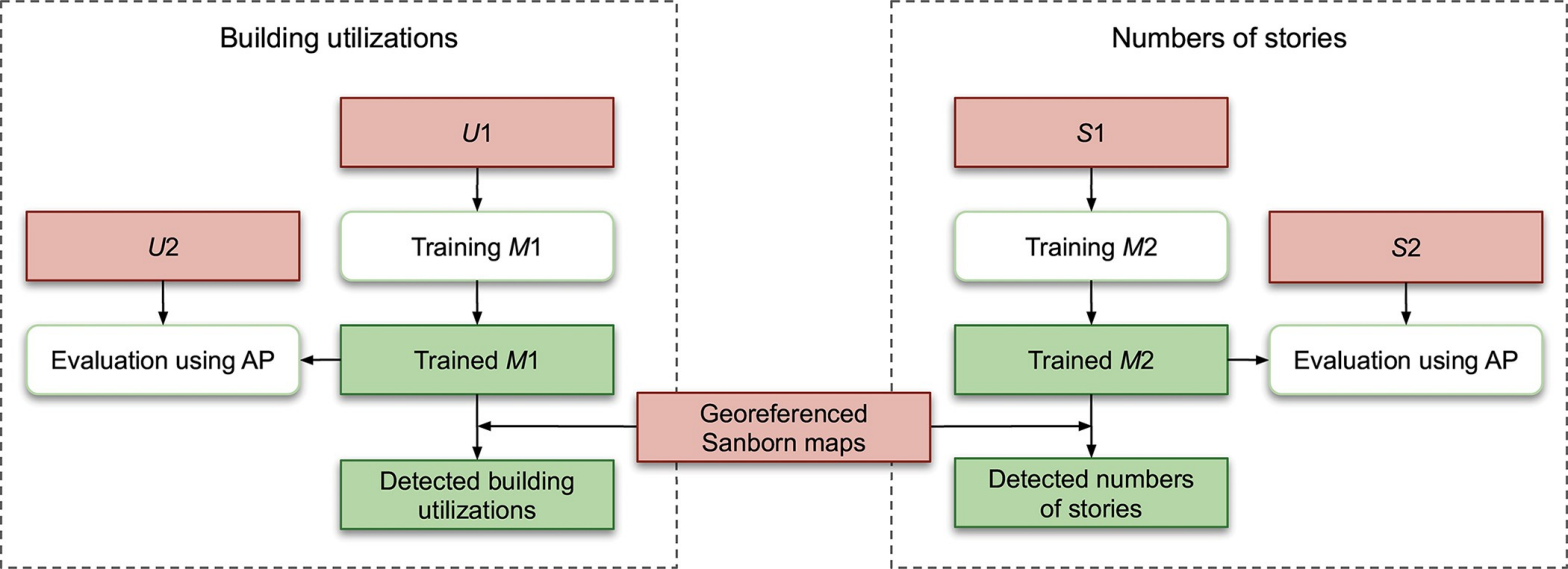
Detecting building utilizations and numbers of stories. An orange-pink rectangle indicates input data, an open rounded rectangle indicates an operation, and a green rectangle represents intermediate or final output.

Fig 6 illustrates the training and evaluation of two machine learning models for detecting building utilizations and numbers of stories. We use data sets *U*1 and *S*1 to train the two machine learning models, *M*1 and *M*2, respectively. We evaluate the trained models on data sets *U*2 and *S*2 using the AP metric. We then apply the trained models to the Sanborn maps to obtain the building utilizations and numbers of stories.

### 3.3 Visualization

Georeferenced Sanborn maps are required for our workflow to extract information to effectively create 3D visualization of historical neighborhoods. Georeferencing is the process of projecting scanned Sanborn maps onto a geographic coordinate system. It requires finding points on the Sanborn map with known geographic coordinates, which are also known as control points. There are different ways to obtain geographic coordinates for control points. A common approach is to match street addresses to records in a database where the coordinates of these locations are known. In addition to street addresses, road intersection can also be used to provide reliable control points [31]. For Sanborn maps, street addresses of buildings are difficult to find their matches in most of the databases that are available today (such as Census TIGER/Line data sets) because the buildings may no longer exist today, but the streets still exist and their intersections can also be matched to the available databases. Once control points are established, a mathematic function can be derived to project the scanned map to a geographic coordinate system so that the difference between the projected control points before and after the projection is minimized [32].

We can visualize the detected building footprints and associated properties (construction materials, utilizations, and numbers of stories) to create 3D digital models of historic neighborhoods. We extrude the detected buildings in proportion to the number of stories, and create façades of the extruded buildings based on their construction materials and utilizations. Specifically, archival images of these neighborhoods are gathered, as well as images from other sources that provide information about the façades of historic buildings. We create façade templates for buildings with different construction materials and utilizations based on the images collected. These façades serve as resources for the visualization of buildings with the same material and utilization in the neighborhoods.

## 4 Application

Hanford Village and Driving Park are adjacent neighborhoods on the Near East Side of Columbus, Ohio. These two neighborhoods once housed a thriving Black community; in the case of Hanford Village, a separate enclave city incorporated in 1909, and developed in 1946 to house returning Black veterans of World War II [77]. During the 1960s, the Federal-Aid Highway Act of 1956 resulted in the construction of Interstate 70 (I-70), which tore apart these two neighborhoods, as well as many others that were home to predominantly Black populations. Today, Driving Park is bisected, and Hanford Village is a remnant of its fuller past. The purpose of this application is to virtually reconstruct the lost neighborhoods of Hanford Village and Driving Park by applying the proposed workflow to historical Sanborn maps.

### 4.1 Data

The Library of Congress has a digital collection of over 25,000 Sanborn maps in more than 3,000 American cities from the 1800s to the present. We retrieved a total of 13 Sanborn maps for Hanford Village and Driving Park depicting the situation in 1961, just before I-70 was built in through these neighborhoods. The retrieved Sanborn maps are approximately 11600×12600 pixels in size. Because our workflow involves pixel-based processing of Sanborn maps, we resampled these maps to 10 percent of their original size (i.e., to roughly 1160×1260 pixels) to reduce the processing time.

We prepared seven data sets—*P*1, *P*2, *O*1, *U*1, *U*2, *S*1, and *S*2— using the Sanborn maps. We used data sets *P*1 and *P*2 in the pixel-based classification of building footprints and construction materials. For each class listed in Table 1, we randomly selected pixels from the 13 Sanborn maps for Hanford Village and Driving Park in 1961 and labeled these pixels with their RGB values. We split the selected pixels into two sets so that we used 90% in *P*1 for model training, and the remaining 10% in *P*2 for model evaluation. Table 3 summarizes the number of pixels for each class in *P*1 and *P*2.

**Table 3:** Number of pixels for each class in data sets *P*1 and *P*2.

Class	Description	Number of pixels		
		*P*1	*P*2	Total
**1**	Brick or tile building	1937	214	2151
**2**	Frame or wood building	1819	219	2038
**3**	Fire resistive building	1696	185	1881
**4**	Adobe, metal, or iron building	1207	136	1343
**5**	Concrete or cinder block building	1858	209	2067
**6**	Non-building: background	2013	210	2223
**7**	Non-building: other symbols or text	1273	139	1412

Data set *O*1 was used to evaluate the accuracy of the postprocessed building objects. We partitioned each of the 13 Sanborn maps into 12 non-overlapping tiles, with a size of approximately 290×420 pixels for each tile. The number of tiles was chosen to ensure adequate coverage of buildings in each tile, which could be used in our test setting. Data set *O*1 contains 20 non-overlapping map tiles from the 13 Sanborn maps. We manually identified and labeled the construction materials of 193 building objects on these map tiles. Table 4 summarizes the number of building objects for each class in *O*1.

**Table 4:** Number of building objects for each class in data set *O*1.

Class	Description	Number of building objects
**1**	Brick or tile building	45
**2**	Frame or wood building	99
**3**	Fire resistive building	6
**4**	Adobe, metal, or iron building	6
**5**	Concrete or cinder block building	37

Data sets *U*1 and *U*2 were used to train and evaluate the model for detecting building utilizations, respectively. For deep neural networks like Mask R-CNN, the machine learning literature generally suggests using a large and diverse training data set to improve detection accuracy [78]. Data set *U*1 thus includes 81 map tiles from the 13 Sanborn maps, as well as another 235 map tiles from 20 Sanborn maps for our study area from 1921. We identified a total of 6776 letters and words that represent different building utilizations from the map tiles included in *U*1. For data set *U*2, we selected 20 non-overlapping map tiles from the 13 Sanborn maps and labeled 378 letters and words. Table 5 summarizes the number of letters and words for each class in *U*1 and *U*2. Note that our study area primarily comprises residential buildings and garages, and as a result, the datasets do not contain commercial and industrial buildings such as post offices and manufacturing facilities.

**Table 5:** Number of letters or words for each class in data sets *U*1 and *U*2.

Class	Description	Number of letters and words		
		*U*1	*U*2	Total
**1**	A	943	114	1057
**2**	Auto	1238	32	1270
**3**	D	4478	230	4708
**4**	F	117	2	119

Finally, data sets *S*1 and *S*2 were used for the detection of numbers of stories. Map tiles in *S*1 and *S*2 are identical as those in *U*1 and *U*2, respectively. We manually labeled the numerals that represent the number of stories of each building in each map tile. Table 6 summarizes the number of numerals for each class in *S*1 and *S*2. Note that our study area exclusively features buildings with 1, 1.5, or 2 stories, and no buildings with three or more stories are present in the data.

**Table 6:** Number of numerals for each class in data sets *S*1 and *S*2.

Class	Description	Number of numerals		
		*S*1	*S*2	Total
**1**	1	10731	512	11243
**2**	1 ½	4884	202	5086
**3**	2	310	13	323

### 4.2 Implementation details

Our workflow starts with georeferencing the 13 Sanborn maps for Hanford Village and Driving Park in 1961. The geocoding service maintained by the Center for Urban and Regional Analysis (CURA) at the Ohio State University was used to establish the control points. The CURA geocoding service uses the address database in the ArcGIS Business Analyst (https://www.esri.com/en-us/arcgis/products/arcgis-business-analyst/overview). More specifically, on each scanned Sanborn map, we manually identified at least 5 road intersections and used CURA geocoding service to obtain their geographic coordinates. With these control points, we used the Georeferencer plugin in QGIS [79] to project the Sanborn maps to the WGS 84 geographic coordinate system.

We then trained the SVM model in Python using the *scikit-learn* library [80] for the pixel-based classification of building footprints and construction materials. Processing each of the 13 Sanborn maps using pixel-based classification took around 20 minutes in our test setting, on a computer equipped with an AMD Ryzen 5600X 6-Core Processor (3.70 GHz) and 32GB RAM. To postprocess the classified pixels, we performed the morphological transformations using a Python library called *cv2* [81]. We used the Raster to Polygon tool in ArcGIS Pro [82] for the vectorization of building pixels. We conducted simplification and regularization of vector building objects using the Simplify Polygon [83] and Regularize Building Footprint [84] tools in ArcGIS Pro, respectively. We detected the building utilizations and numbers of stories using the Mask R-CNN models implemented with the source code released by [85] on a remote server with two NVIDIA Tesla P100 GPUs, with the models running approximately 60 seconds per map.

We performed manual check and edits to the detected building-level information based on results of the accuracy assessment (details of the assessment in the Accuracy Assessment section). The manually checked information was used to create a 3D digital model of the two neighborhoods. Archival panoramic images of Hanford Village and Driving Park in the 1920s were from the Blanchard Photo Collection [86]; this helped us inform facades of buildings with different construction materials and utilizations at the time period. We obtained additional images from Zillow (https://www.zillow.com/) to reflect different building facades, with buildings constructed before or around the 1960s being chosen. Finally, we used a 3D modeling software called ArcGIS CityEngine (www.esri.com/cityengine) to create a 3D digital model of Hanford Village and Driving Park based on the facade and number of stories of each building.

### 4.3 Accuracy assessment

**Fig 7:**
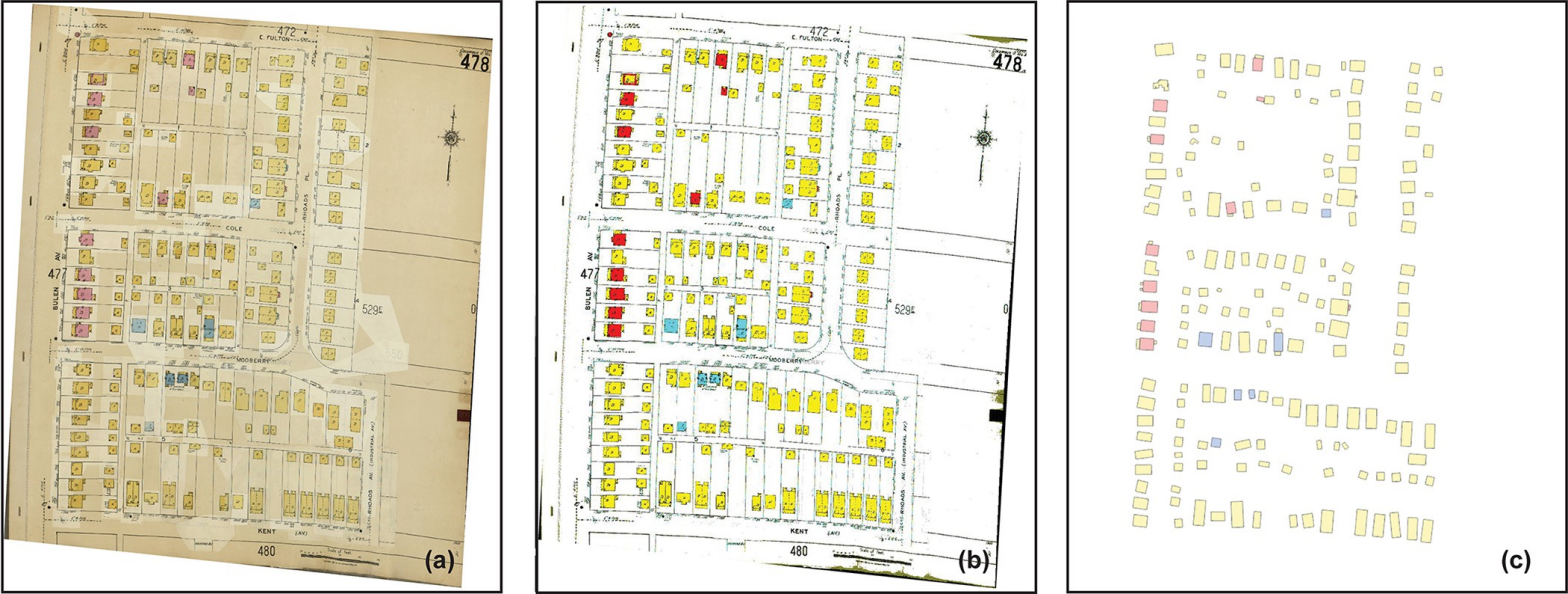
Detected building footprints and their construction materials on a georeferenced Sanborn map. (a) is a georeferenced Sanborn map, (b) shows the results of pixel-based classification, and (c) presents the vector building objects after post-processing. The colors used in the figure represent the different construction materials of the buildings, and their meanings can be found in Table 1.

Fig 7 shows the detected building footprints and construction materials on a sample Sanborn map for our study area. Visual analysis of the results reveals that the detected building footprints and construction materials, both pixel-level and object-level, align well with the those on the original Sanborn map. In addition, despite the noise of text and symbols within and around buildings on the Sanborn map, our workflow ensures that the final building objects (Fig 7C) are of realistic shape and do not deviate from the original.

Table 7 shows results of the pixel-level accuracy evaluation for the detected building footprints and construction materials. For classes 1 and 2, the precision, recall, and F-score all have values of 1, indicating that all detections are correct and that there are no missing building pixels. Classes 3 and 5 have precision and recall values higher than 0.85, which means that over 85 percent of the building pixels in these two classes can be correctly identified, and over 85 percent of the classified pixels are correct. These results suggest high accuracy in the detections of pixels in classes 3 and 5. Class 4 has a high recall of 0.9 and a precision of 0.8. In practice, the slightly low precision value would imply inspecting the classified pixels in class 4 more carefully to determine whether they belong to other classes (e.g., background). In addition to the three metrics of precision, recall, and F-score, we calculate the average for each metric over all classes, weighted by the number of pixels or objects in each class [87]. The weighted average provides information about the overall accuracy across all classes. The results show that the weighted averages for precision, recall, and F-score are all above 0.9, indicating a high level of accuracy for the pixel-based classification in general.

**Table 7:** Results of the pixel-level accuracy evaluation.

Class	Description	Precision	Recall	F-score
**1**	Brick or tile building	1.00	1.00	1.00
**2**	Frame or wood building	1.00	1.00	1.00
**3**	Fire resistive building	0.87	0.94	0.90
**4**	Adobe, metal, or iron building	0.80	0.90	0.85
**5**	Concrete or cinder block building	0.99	0.86	0.92
**Weighted average**		**0.94 **	**0.93 **	**0.93 **

Table 8 shows results of the object-level accuracy evaluation for the detected building footprints and construction materials. Classes 1, 2, 4, and 5 all have precision above 0.8, implying that more than 80 percent of the building objects categorized to be in these classes are correct. Class 3, with a precision of 0.75, is likely to require additional attention to determine whether a building object categorized in this correct class. The recall for all classes is above 0.8, indicating that the majority of building objects in each class are correctly identified. The weighted averages for precision, recall, and F-score are computed, and their values are all above or close to 0.9. This indicates overall accurate detections of buildings at the object level.

**Table 8:** Results of the object-level accuracy evaluation.

Class	Description	Precision	Recall	F-score
**1**	Brick or tile building	1.00	1.00	1.00
**2**	Frame or wood building	0.91	0.81	0.86
**3**	Fire resistive building	0.75	1.00	0.86
**4**	Adobe, metal, or iron building	0.83	0.83	0.83
**5**	Concrete or cinder block building	0.85	0.95	0.90
**Weighted average**		**0.91 **	**0.89 **	**0.90 **

**Fig 8:**
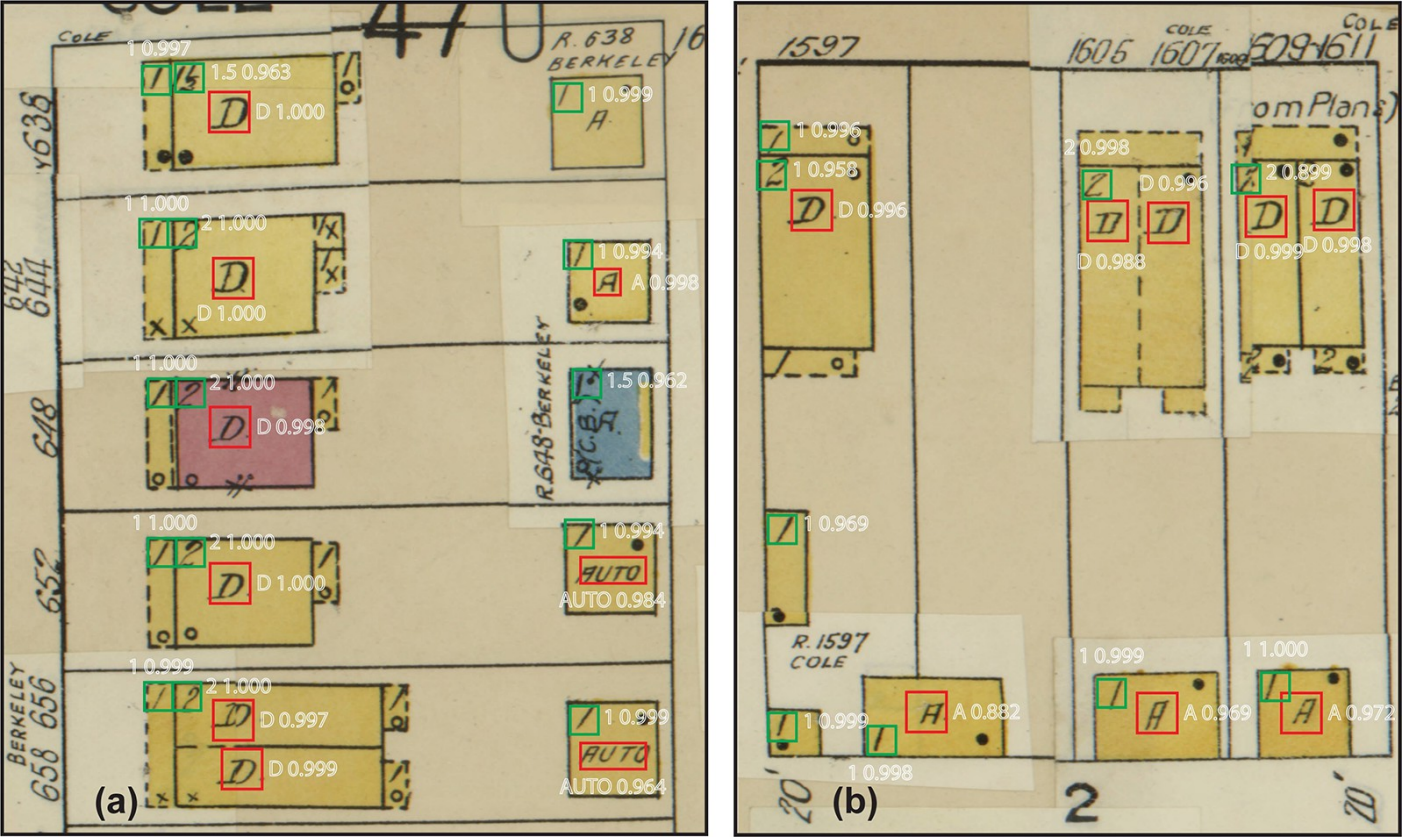
Detected building utilizations (bounding boxes in red) and numbers of stories (bounding boxes in green) on Sanborn maps. The text in white includes a detected abbreviation that denotes a building utilization or the number of stories, as well as a confidence score of the detection.

Fig 8 illustrates the detected building utilizations and numbers of stories. The detection results include a class label (e.g., abbreviations “A”, “D” or “1”, “2”), a bounding box (hollow rectangles in red or green), and a confidence score of the detection. Most of the detections have confidence scores above 0.9, indicating that the models perform well in detections. Visual analysis of the detection results reveals that the detected building utilizations and numbers of stories well match those on Sanborn maps. The trained models effectively distinguish the target abbreviations that denote building properties from other text, such as block numbers.

Table 9 presents results of the accuracy evaluation for the detected building utilizations. The average precision (AP) for classes 3 and 4 are above 0.95, indicating that more than 95 percent of the detected building utilizations in these two classes are correct. Classes 1 and 2, which represent the building utilization as garage, have slightly low AP values of 0.84 and 0.8, respectively. This means that buildings identified as garages would require manual verification to ensure that the detections were correct. We compute the weighted average of AP across all classes, weighted by the number of abbreviated letters or words in each class. The resulting weighted average is 0.92, indicating a generally high level of accuracy.

**Table 9:** Results of the accuracy evaluation for the detected building utilizations.

Class	Description	AP
**1**	A	0.84
**2**	Auto	0.80
**3**	D	0.97
**4**	F	1.0
**Weighted average**		**0.92**

Table 10 presents results of the accuracy evaluation for the detected numbers of stories. The AP ranges between 0.65 and 0.8 for all three classes of abbreviated numerals that denote the numbers of stories. The AP values are lower than those for building utilizations, but they still indicate that most of the detected numbers of stories are accurate. The weighted average of AP over all classes is 0.72, suggesting that 72 percent of the detections are correct.

**Table 10:** Results of the accuracy evaluation for the detected numbers of stories.

Class	Description	AP
**1**	1	0.69
**2**	1 ½	0.79
**3**	2	0.77
**Weighted average**		**0.72**

### 4.4 A 3D digital model of the lost neighborhoods of Hanford Village and Driving Park

**Fig 9:**
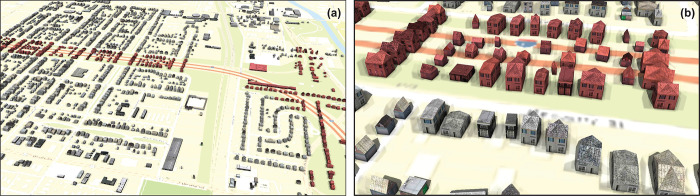
Reconstructed historic neighborhoods of Hanford Village and Driving Park. I-70 is colored orange, and the buildings that have been demolished are red.

Fig 9 presents a 3D digital model of the historic neighborhoods of Driving Park (left side of Fig 9A) and Hanford Village (right side of Fig 9A) based on building footprints and associated properties extracted from Sanborn maps. Fig 9B shows a close up of the building objects. An interactive visualization can be found at https://bit.ly/3Dj3IgN. Both Figs also show contemporary I-70, built afterwards. We identified the demolished buildings due to I-70 construction by comparing the building footprints in 1961 and the current built-up areas in the neighborhoods; these are the red-tinted buildings. The results show that a total of 380 buildings have been demolished in these areas, including 286 dwellings, 86 garages, 5 apartments, and 3 stores.

## 5 Discussion and conclusions

The extensive historical archive of map atlases [10], gazetteers [88], and literature [89] has been a valuable resource for understanding what our cities were like in the past. Over the last two decades, emerging computational methods have provided opportunities for a thorough, comprehensive, and integrated analysis of urban history through the study of the humanities [90, 91]. This paper presents a scalable computational workflow that enables automated geographic information extraction to inform urban changes from historical maps, specifically, Sanborn Fire Insurance maps that contain a wealth of building-level data for thousands of U.S. cities from the late 19th to late 20th centuries. Our experimental results show that our workflow is effective at extracting information with high accuracy and creating realistic 3D digital models of historic urban neighborhoods. This research is an essential step toward exploring and demonstrating the potential of computational methods for urban studies within and beyond the humanities.

The proposed workflow has the potential to be applied to other geographic areas and time periods, albeit depending on the availability of the Sanborn maps and software and computational resources. Our computational workflow requires the digital map scans to be in color, as the methodology for detecting building footprints and construction materials relies on pixel colors and cannot be directly applied to the black-and-white map scans. Fortunately, the Library of Congress has a vast collection of approximately 700,000 digital map scans covering over 12,000 American cities and towns dating from 1867 to present, which is a suitable source for this purpose. This extensive data source provides ample opportunity to generalize the application of our workflow beyond the two neighborhoods and year studied. There are also other sources of Sanborn maps digitalized from microfilm collections and are in black and white, such as those from ProQuest (https://about.proquest.com/en/products-services/sanborn/). Since building construction materials on the black-and-white maps are marked as letters like what has been done for building utilizations, one possible solution to leveraging these black-and white maps is to first use computer vision techniques, such as edge detection [92], to extract the building footprints, and then develop text detection models, similar to what we have done for building utilizations, to classify the construction materials of the buildings. This would enable us to make use of a broader resource of digital Sanborn maps.

Creating 3D visualizations using the proposed framework requires georeferenced Sanborn maps, which can often be obtained from established sources like ProQuest or through manual georeferencing. Recent advancements in automated georeferencing have also made it possible to derive these maps efficiently. For example, research on automated identification of landmarks [93] and road intersections [33] makes it possible for efficient control points retrieval. New geocoding methods, such as historical collaborative geocoding [94, 95] and deep learning-based geocoding [96] techniques, are being developed, along with open source geocoding tools such as the Historical Geocoding Assistant [97]. Automated georeferencing methods have been developed to align historical maps with online reference data from sources such as OpenStreetMap [27, 98–100]. A combination of these research outcomes is promising to enable highly accurate automated georeferencing.

The proposed workflow relies partially on existing software, such as ArcGIS Pro and QGIS, and computational resources, such as GPU. While the reliance on ArcGIS Pro and QGIS is relatively simple and involves only a small part of the entire workflow, their automation can be achieved using recent advancements in open-source Python libraries such as *shapely* [101] and *rasterio* [102] that offer various functions for efficient building vectorization and simplification. Integrating these software libraries into our workflow can also significantly improve the automation of the proposed workflow and enhance generalizability. In this study, GPU is used to support the training of deep learning Mask R-CNN model. Online platforms, such as Google Colaboratory (https://colab.research.google.com/), provide free access to GPU, which can eliminate the barriers to computational resources and help generalize our workflow.

Sanborn maps often include the names and detailed descriptions of industrial and institutional buildings, such as manufacturing plants, schools, churches, and hospitals. This textual information makes an excellent resource for tracing the evolution of facilities as well as the history of a city. Although our proposed approach is not intended to detect detailed textual data for industrial and institutional buildings, it is possible to supplement our existing training data with labeled detailed textual data and investigate other state-of-the-art models for text detection [103, 104] and recognition [105] in the future. This will help generalize our methods to areas dominated by industrial and institutional buildings.

Sanborn maps also provide a wealth of property information, such as property boundaries and street addresses, which merits further investigation. One important application of this information is to use it along with building footprints and auxiliary data, such as the city directories, for estimating the economic loss caused by the demolition of historic neighborhoods. Research has demonstrated the potential of using computational methods to identify and match markers and labels on historical maps [106]. This allows us to combine the property information on Sanborn maps and the building information derived using our existing framework. Future research can be directed toward expanding on this line of inquiry in order to fully utilize the urban information available from Sanborn maps.
